# COVID-19 Vaccination and the Rate of Immune and Autoimmune Adverse Events Following Immunization: Insights From a Narrative Literature Review

**DOI:** 10.3389/fimmu.2022.872683

**Published:** 2022-07-05

**Authors:** Naim Mahroum, Noy Lavine, Aviran Ohayon, Ravend Seida, Abdulkarim Alwani, Mahmoud Alrais, Magdi Zoubi, Nicola Luigi Bragazzi

**Affiliations:** ^1^ International School of Medicine, Istanbul Medipol University, Istanbul, Turkey; ^2^ Zabludowicz Center for autoimmune diseases, Sheba Medical Center, Ramat-Gan, Israel; ^3^ St. George School of Medicine, University of London, London, United Kingdom; ^4^ Laboratory for Industrial and Applied Mathematics (LIAM), Department of Mathematics and Statistics, York University, Toronto, ON, Canada

**Keywords:** COVID-19 vaccine, vaccine hesitancy, autoimmune side effects, Guillain-Barre syndrome, myocarditis, vaccine-induced immune thrombotic thrombocytopenia

## Abstract

Despite their proven efficacy and huge contribution to the health of humankind, vaccines continue to be a source of concern for some individuals around the world. Vaccinations against COVID-19 increased the number of distressed people and intensified their distrust, particularly as the pandemic was still emerging and the populations were encouraged to be vaccinated under various slogans like “back to normal life” and “stop coronavirus”, goals which are still to be achieved. As fear of vaccination-related adverse events following immunization (AEFIs) is the main reason for vaccine hesitancy, we reviewed immune and autoimmune AEFIs in particular, though very rare, as the most worrisome aspect of the vaccines. Among others, autoimmune AEFIs of the most commonly administered COVID-19 vaccines include neurological ones such as Guillain-Barre syndrome, transverse myelitis, and Bell’s palsy, as well as myocarditis. In addition, the newly introduced notion related to COVID-19 vaccines, “vaccine-induced immune thrombotic thrombocytopenia/vaccine-induced prothrombotic immune thrombotic thrombocytopenia” (VITT/VIPITT)”, is of importance as well. Overviewing recent medical literature while focusing on the major immune and autoimmune AEFIs, demonstrating their rate of occurrence, presenting the cases reported, and their link to the specific type of COVID-19 vaccines represented the main aim of our work. In this narrative review, we illustrate the different vaccine types in current use, their associated immune and autoimmune AEFIs, with a focus on the 3 main COVID-19 vaccines (BNT162b2, mRNA-1273, and ChAdOx1). While the rate of AEFIs is extremely low, addressing the issue in this manner, in our opinion, is the best strategy for coping with vaccine hesitancy.

## Introduction

Considered one of the fastest processes of manufacturing vaccines ever, “Coronavirus Disease 2019” (COVID-19) vaccines were discovered, studied, and produced in terms of months since the declaration of COVID-19 as a pandemic by the World Health Organization (WHO) (1). While the burden of the outbreak alongside the support of the governments were the main motivations for the accelerated vaccine production, the process itself aroused concerns regarding the safety of the COVID-19 vaccines and contributed to mistrust in health authorities as well as in the vaccines themselves (2, 3). Interestingly, distrust in COVID-19 vaccines was reported even before the vaccines were available (4). It is of great importance to mention that vaccines are highly effective in reducing the burden of infectious diseases as the history clearly shows (5, 6), and the COVID-19 pandemic is likely to follow a similar trend after the roll-out of the COVID-19 vaccination has been fully implemented, globally. However, the fear of the lay public for adverse events following immunization (AEFIs), especially those of severe and long-term features, may constitute the main obstacle in successfully fighting against COVID-19 vaccine hesitancy (7, 8). This is especially true for autoimmune and immune-mediated AEFIs such as idiopathic thrombocytopenia (ITP), or immune thrombocytopenia, Guillain-Barré syndrome (GBS), and transverse myelitis (TM), among others. Therefore, it is crucial to address the mounting concern by reviewing the past, present, and possible future AEFIs of vaccines in general and COVID-19 vaccines in particular, especially as millions were already vaccinated around the world, and more data has accumulated regarding the safety profile of the COVID-19 vaccines (9). Addressing AEFIs of autoimmune nature covers, to a great extent, the concerns generated by the public explicitly considering vaccine booster programs endorsed by the health authorities. The importance of facing and addressing public concerns in dealing with vaccine hesitancy cannot be overemphasized (10).

We hereby present a detailed, narrative literature review covering the various types of vaccines, their immune-mediated AEFIs, and we focus on the autoimmune AEFIs described in correlation with the main COVID-19 vaccines in use worldwide, that is to say, those most administered (approximately 95% of those delivered). The rarity of the AEFIs among vaccinated people, and the risk of severe complications that COVID-19 carries, are the key factor for fighting against vaccine hesitancy.

## Types of Vaccines, Adverse Events Following Immunization, and COVID-19 Related Vaccines

Vaccines that were approved or in use against COVID-19 can be classified as follows:

### Inactivated Vaccines

Inactivated or killed vaccines contain a pathogen that has been inactivated after being grown in a culture. The first utilized was the cholera vaccine introduced in 1896 (11). There are two subtypes of inactivated vaccines: the traditional whole-cell vaccine and the acellular vaccine. The latter is a more modern version and contains only 3-4 antigens rather than the hundreds of microbial antigens present in the whole-cell vaccine. This antigen reduction produces a more specific immune response, thereby reducing potential AEFIs (12), such as febrile responses (13). While traditional whole virion-inactivated influenza vaccines and whole cell pertussis vaccines commonly cause febrile responses within 24 hours of immunization, the split virion influenza vaccine and the acellular pertussis vaccine showed a decreased rate of febrile illness (14, 15). Concerning inactivated vaccines against COVID-19, the vaccine developed by the Beijing Institute of Biological Products, known as BBIBP-CorV, and referred to as Sinopharm, is an inactivated vaccine. In a randomized, double-blind, dose-escalation, controlled phases I and II trial of the BBIBP-CorV vaccine, an acceptable safety profile and a robust humoral response to the coronavirus were reported (16). The study was conducted among 18-80 years old, healthy individuals negative for serum-specific IgM or IgG antibodies against both N and S proteins of the virus. The inactivated BBIBP-CorV vaccine was immunogenic and elicited strong humoral responses, with a 100% seroconversion rate in all groups. However, lower seroconversion rates were found in the group of individuals aged 60 and older, probably due to the atrophy/ageing of the immune system (17). The degree of local and systemic AEFIs was generally mild and occurred most after the first dose of vaccination (18).

### Viral Vector-Based Vaccines

Viral vectors were first introduced in relation to gene and cancer therapy but, since then, have been adapted to vaccine development (19). The utility of vector-based vaccines is determined by the capacity of viruses to infect cells (20). High efficiency gene transduction, extremely selective gene delivery to target cells, generation of powerful immunological responses, and improved cellular immunity are the advantages of viral vector-based vaccines (20). Multiple viruses ranging from very complex large DNA viruses such as poxviruses, to simple RNA viruses such as parainfluenza virus, have been deployed as viral vectors (21). Viral vector vaccines facilitate intracellular antigen expression and trigger a highly cytotoxic T-lymphocyte response (22). A significant emphasis was made on COVID-19 vaccines based on viral vectors like the AstraZeneca, Janssen/Johnson & Johnson, and CanSino vaccines (19), which are based on adenoviruses (23). In addition to the vaccines listed above, Sputnik V, also known as Gam-COVID-Vac, is a COVID-19 adenovirus viral vector vaccine (24). Injection site pain is considered the most common local AEFI of viral vector vaccines, together with headache, fatigue, muscle pain, malaise, chills, and joint pain (25).

### mRNA Vaccines

Because of their high potency, ability to evolve quickly, and potential for low-cost manufacturing and safe delivery, mRNA vaccines were found to be a promising alternative to traditional vaccine techniques. In 1990, the first effective application of *in vitro* transcribed (IVT) mRNA in animals was described, when reporter gene mRNAs were injected into mice and protein synthesis was detected (26). However, this finding did not lead to an increase in the mRNA vaccine investment, due to concerns regarding mRNA instability, inefficient *in vivo* delivery, and high innate immunogenicity. Subsequently, over the past decade, the use of mRNA vaccines has been gradually implemented due to their low potential risk of infection and insertional mutagenesis (27). Actually, the concept behind the mRNA vaccine is to deliver the mRNA of the pathogen into the human body in order to trigger an immune response and produce antibodies against the pathogen. There are two types of mRNA vaccines available for prevention of infectious diseases: self-amplifying or replicon RNA vaccines (SAM) and non-replicating mRNA vaccines. SAM vaccines have the benefit of producing their own adjuvants in the form of dsRNA structures, replication intermediates, and other motifs, which may explain their high potency. Directly injectable, non-replicating mRNA vaccines are promising vaccination products due to their convenient way of administration and low cost, especially in resource-constrained environments (28). Two of the most widely distributed COVID-19 vaccines, namely the BNT162b2 (BioNTech/Pfizer) and the mRNA-1273 (Moderna), are mRNA-based vaccines. The BNT162b2 vaccine, for instance, proved to be around 95% efficient in preventing the disease, with a relatively high safety profile (29). However, mild and short-term AEFIs were reported such as pain at the injection site, fatigue, and headache. The incidence of serious AEFIs was low.

## Roll-out of COVID-19 Vaccines

As herd immunity is achieved once a large portion of the population is immune against an infectious agent preventing it from widely spreading, it is a must in overcoming the COVID-19 pandemic (30). In addition, herd immunity is also necessary to protect individuals who are unable to get vaccinated such as infants, children under the recommended age, and immunocompromised individuals (31). In turn, people become immune against a specific infection in two ways, either through acquiring the infection naturally from a pathogen, or passively by vaccination. While COVID-19 can be severe and fatal, especially in people at risk such as the elderly, those with chronic cardiovascular and respiratory disease, among others (32), vaccinations is paramount in terms of preventing disease acquisition as well as its spread. The US Food and Drug Administration (FDA) authorized the first vaccine for COVID-19 on December 11^th^, 2020 (33). More specifically, the first vaccine authorized was BNT162b2 (BioNTech/Pfizer COVID-19 vaccine) under the Emergency Use Authorizations (EUA). The United Kingdom (UK) Medicines and Healthcare products Regulatory Agency (MHRA) had already authorized the use of the same vaccine, some days before, on December 2^nd^, 2020 (34). Subsequently, the European Union (EU) commission authorized the vaccine on December 21^st^, 2020 (35). Shortly afterward, the mRNA-1273 COVID-19 vaccine (Moderna) was authorized (36). Later on, the ChAdOx1 vaccine (AstraZeneca) was authorized as well by the UK MHRA (37). The three mentioned vaccines were the most commonly used vaccines across the globe. By the end of November, 2021, approximately 55% of the world population has received at least one dose of a COVID-19 vaccine, 7.9 billion doses have been given globally, and around 27 million new doses are administered daily (38). Asia had the most doses administered at 5.09 billion doses, followed by Europe with 942 million doses, North America with 739.54 million doses, South America with 581.31 million doses, Africa with 235.45 million doses, and finally Oceania with 49.67 million doses.

## Autoimmune Adverse Events Following Immunization and Their Correlation With Vaccines in General and COVID-19 Vaccines in Particular

### Autoimmune Neurological Adverse Events Following Immunization

#### Guillain-Barre’ Syndrome (GBS)

GBS is an autoimmune disorder characterized by an immune-mediated nerve damage generally triggered by an infectious agent leading to cross-reactive antibodies attacking axonal antigens and resulting in demyelinating polyneuropathy (39). Despite its rarity, GBS is considered as the most common cause of acute flaccid paralysis worldwide (40), with about 100,000 people developing the disorder every year (41). The disease is manifested by ascending and symmetrical flaccid paralysis leading to paresthesia, autonomic dysfunction, and respiratory muscle paralysis (42). In some instances, cranial nerve involvement is also seen, causing facial diplegia (43). The complications of GBS can be fatal, including respiratory and cardiac failure (44). The mortality rate of GBS varies widely from 1-18% (45, 46). Numerous reports have suggested a possible relationship between GBS and vaccines. However, solid evidence was not established (47, 48). During the COVID-19 pandemic, various reports addressed the correlation between COVID-19 and GBS; nevertheless, a concrete causal relationship is yet to be established (40).

##### BNT162b2 (BioNTech/Pfizer) and GBS

Several case reports were published regarding GBS following the BNT162b2 vaccine. A 82-years-old female presented two weeks following her first dose (49), and a 67-year-old male seven days after the first shot (50). In addition, a 71-year-old male with Miller-Fisher syndrome (MFS), a rare variant of GBS, presented 18 days following his first dose of BNT162b2 vaccine (22). GBS was also documented following the second dose of the vaccine. For instance, a 25-year-old female developed a clinical picture of GBS few days following her second dose of the vaccine, and a 73-year-old male twenty days following the second dose of the vaccine (51). Furthermore, Ben David and colleagues (52) conducted a retrospective cohort study aimed to assess the safety of mRNA-based COVID-19 vaccine in previously diagnosed cases of GBS between 2000-2020. Based on a database from a health organization serving more than 2.5 million members, the authors found that out of 702 members who had a diagnosis of GBS, 579 received at least one vaccine dose, and only one patient presented with a relapse of GBS several days following the second dose, which represents a minimal risk. In addition, Shasha et al. (53) investigated AEFIs profile following BNT162b2 vaccine in a sample size of over 400,000 individuals, and found that only one individual in the vaccinated group had GBS versus none in the control group. Similarly, seven cases of GBS were reported following the first dose of BNT162b2 mRNA vaccine among approximately 4 million recipients (incidence of 0.18/100,000), while no cases recorded following the second dose. The study was conducted over a period of 30 days following vaccination concluding that among recipients of the BNT162b2 mRNA vaccine, GBS may occur at the expected community-based rate (54).

##### mRNA-1273 (Moderna) and GBS

To the best of our knowledge, only two case reports have been published regarding GBS following Moderna, mRNA-1273 COVID-19 vaccine. Both of the cases developed after the second dose. In the first case (55), the symptoms appeared two days after vaccination presented by an axonal variant of GBS. In turn, in the second case (56), the symptoms appeared 6 weeks following the vaccination, while the electrophysiological test identified a mixed axonal and demyelinating type of GBS. Furthermore, among 16 cases of acute-onset polyradiculoneuropathy that presented within 4 weeks after first dose of SARS-CoV2 vaccines, only one person received the mRNA-1273 vaccine, whereas 14 received the ChAdOx1 vaccine (57).

##### ChAdOx1 (AstraZeneca) and GBS

During July 2021, the European Medicines agency (EMA) recommended that GBS should be added as a warning sign on the ChAdOx1 vaccine product information, despite being unable to decisively conclude about a causal association (58). This action was in part due to multiple reports of GBS cases occurring within a month following the first dose of the vaccine (59–67). The series of cases caused a major concern among leading experts. In fact, the GBS cases reported after the ChAdOx1 vaccine often presented as facial diplegia and paresthesia, a rather rare manifestation of the condition, with clinical improvement after corticosteroid and intravenous immune globulins (IVIG) therapy. The cases had no known exposure to the coronavirus and tested negative upon admission. However, as a recent study found that the COVID-19 infection was unlikely to cause GBS (68), the significance is debatable. Other baseline characteristics, such as age, gender and associated morbidity differed between cases, and scholars are therefore unable to make a clear-cut connection between them. The incidence of GBS was estimated to be approximately 0.89 to 1.89 cases per 100,000 person-years (69). A recent report issued by the UK Health Security Agency concluded that the risk of developing GBS after the first dose of ChAdOx1vaccine adds 5.6 extra cases of GBS per million doses, while having no association with respect to the second dose (70). As the condition has also been linked to the Ad26.COV2.S (Janssen/Johnson & Johnson) COVID-19 vaccine (71), another adenoviral vector vaccination, further investigations into the pathogenesis are required as large studies about GBS rate are lacking, and conclusions should not be drawn without further analysis.

#### Transverse Myelitis

TM is an immune-mediated acute or a subacute inflammatory disease of the spinal cord accompanied with motor, sensory, and autonomic symptoms (72). The clinical presentation of TM varies depending on the level of the spinal cord involved as the disease is manifested below the affected segment. Patients presenting with TM can be paraplegic, most having urinary bladder function disorders and paresthesia (73). The exact etiology of TM has not been established yet. Nevertheless, different types of vaccines were formerly linked to the appearance of TM including hepatitis B vaccine, MMRV and others (74). Most of the cases documented occurred between several days to several months from vaccination however, longer durations were also recorded.

##### BNT162b2 (BioNTech/Pfizer) and Transverse Myelitis

Actually, little evidence with no obvious association exists regarding the appearance of TM after the administration of BNT162b2 COVID-19 vaccine. Case reports include a 75-year-old Japanese patient who presented with TM 3 days following the first dose of the BNT162b2 vaccine (75). The authors could not elucidate a clear association between the vaccine and the clinical presentation and concluded that more epidemiological studies are needed. Furthermore, among more than 700,000 individuals who received the first dose of the BNT162b2 vaccine in Mexico, only 2 cases of TM were documented, equal to a rate of 0.28 per 100,000 cases (76).

##### mRNA-1273 (Moderna) and Transverse Myelitis

Concerning the mRNA-1273 COVID-19 vaccine, case reports of ADEM (77), neuromyelitis optica (78), and acute TM (79) were reported. Ismail et al. (80) reviewed central nervous system (CNS) demyelination disorders among recipients of various COVID-19 vaccinations. A total of 32 cases were registered. Among the cases, higher rates of women (68.8%) than men and a median age of 44 years were noticed. Moreover, most of the cases (71.8%) occurred following the first dose whereas more than a half of the cases had a previous history of immune-mediated diseases (53.1%). As for TM in particular, 6 cases occurred after receiving the mRNA-1273 vaccine. According to the same study, the other vaccines possibly correlating with TM were as follows: 11 cases following BNT162b2 vaccine, 8 after ChAdOx1 vaccine, 5 after Sinovac/Sinopharm vaccines, and one after each of the Sputnik and the Janssen/Johnson & Johnson vaccines.

##### ChAdOx1 (AstraZeneca) and Transverse Myelitis

During the phase 3 clinical trial of the ChAdOx1 vaccine, 3 participants were diagnosed with TM (81). As a result, the trial was temporarily paused enabling further investigations. Two of the cases were determined to be unrelated to the vaccine, the first one had pre-existing, undiagnosed multiple sclerosis; and the other was in fact in the control group with his symptoms appearing over 68 days post vaccination (82). As experts concluded the phenomenon was unlikely to be related to the vaccination the trial resumed however, the incidents raised global concerns regarding the safety profile of the ChAdOx1 vaccine. The longitudinally extensive TM (LETM) is a rare subtype of TM, in which the damage extends over 3 or more vertebrae (83). Reports regarding the LETM subtype started to emerge following the worldwide distribution of the COVID-19 vaccines (84–88). All cases occurred in patients under 60 years of age who presented with neurological symptoms starting within 3 weeks of receiving the first dose of the ChAdOx1 vaccine. As the COVID-19 infection was previously implicated in inducing acute TM (89), all reported cases tested negative on standard PCR testing. Furthermore, all cases completely recovered after treatment with either high dose corticosteroids or plasma exchange and were subsequently discharged.

#### Bell’s Palsy

Bell’s palsy, or facial nerve palsy, is the partial (paresis) or total (paralysis) loss of function of the facial nerve (90). The etiology of Bell’s palsy is most commonly idiopathic but may occur due to several factors such as viral infections (herpes viruses), ischemia, inflammatory and immune-mediated diseases (91). Bell’s palsy is divided into central facial palsy and peripheral facial palsy (92). Central palsy is characterized by contralateral sensory disturbances, as well as dry mouth. In turn, peripheral palsy is characterized by ipsilateral paralysis of the eyelid and forehead muscles.

In a case-control study from Switzerland which aimed to assess the correlation between the inactivated intranasal influenza vaccines and Bell’s palsy (93), a significantly increased risk of Bell’s palsy was found among vaccinated people. The risk of developing Bell’s palsy was 19 times higher than the control group. As a result, the vaccine was withdrawn from clinical use.

##### BNT162b2 (BioNTech/Pfizer) and Bell’s Palsy

The safety database of the BNT162b2 vaccine revealed a slight increase in the cases of Bell’s palsy in vaccinated individuals (94). There were 4 cases of Bell’s palsy in the vaccine group compared to none in the placebo group. As the rate was as expected in the general population, no causal relationship was established. Reviewing the reported safety data concluded that mRNA-based vaccines might be associated with Bell’s palsy (95). Accordingly, several case reports highlighted a similar association, including a healthy 37-year-old male who had Bell’s palsy several days following the first BNT162b2 vaccine dose (96). In addition, different population-based studies which investigated the adverse effects of the BNT162b2 vaccine reported an increase in the frequency of Bell’s palsy as well. For instance, in a nationwide study from Israel which analyzed more than 800 thousand people, the incidence of Bell’s palsy was higher in the vaccinated group compared to the control group, but without significant results (97). Similarly, Shibli and colleagues (98) researched a database of Bell’s palsy cases occurring within 21-days after the first dose and 30-days after the second dose of the vaccine in comparison to the expected cases based on a database from 2019. The authors found a slightly increased incidence of Bell’s palsy following the first dose, mainly among females aged 65 and older, with an estimated attributable risk of 4.46 per 100,000 vaccinated individuals. The study suggested an association between the vaccine and an increased risk of Bell’s palsy but with a small impact on public health. In contrast, Shasha et al. (53) reported no association between COVID-19 vaccines, including BNT162b2, and Bell’s palsy among more than 400 thousand vaccinated people compared to the control group.

##### mRNA-1273 (Moderna) and Bell’s Palsy

Three cases of Bell’s palsy were reported following vaccination with mRNA-1273 vaccine (99–101). The symptoms appeared 12 hours to 2 days after the vaccine was administered. Two cases occurred after the first dose, while one appeared following the second dose. In one patient, a prior episode of Bell’s palsy was recorded whereas the other 2 persons were healthy young people aged 35-36-year-old. Moreover, Sato et al. (102) showed that the rate of Bell’s palsy after both types of the mRNA COVID-19 vaccines (BNT162b2 and mRNA-1273) are lower or equivalent to the rate of Bell’s palsy after influenza vaccines. Additionally, a study from Singapore including 1.4 million subjects who received COVID-19 vaccination, 86.7% by BNT162b2 vaccine and 13.3% by mRNA-1273 vaccine, 11 patients were referred to hospital with Bell’s palsy and 27 patients had cranial mononeuropathy (103). According to data from the WHO pharmacovigilance database, no close association between BNT162b2 and mRNA-1273 COVID-19 vaccines and facial paralysis could be found (104).

##### ChAdOx1 (AstraZeneca) and Bell’s Palsy

While Bell’s palsy is a lower motor neuron disease manifesting as a unilateral facial paralysis (105), GBS may also present with facial paralysis, and most cases linked to the ChAdOx1 vaccine presented in this form (59–67). Therefore, it is difficult to distinguish between the two conditions. One study attempted to examine the risk of co-occurrence of Bell’s palsy and GBS following COVID-19 vaccinations and found an increased risk of co-occurrence following the ChAdOx1 vaccination (106). However, as previously mentioned, the study could not distinguish between the two conditions. Furthermore, a review of published literature failed to identify case reports of isolated Bell’s palsy linked to the ChAdOx1 vaccination. Therefore, it is impossible to draw conclusions of a possible link.

#### Encephalitis

Postvaccinal encephalitis was described in regard to COVID-19 vaccines. In a case series of three patients who presented with symptoms suspected of encephalitis in a range of 7-11 days of receiving the ChAdOx1 vaccine was previously documented (107). The symptoms included gait disturbance, aphasia, headaches, and seizures. As the criteria for autoimmune encephalitis was fulfilled in the three cases, treatment with systemic corticosteroids led to clinical improvement. Other causes of encephalitis including infectious agents were ruled out. All cases were mild and resolved without sequelae. Due to its rarity, the authors highlighted that the benefits of the vaccine outweigh the risks. It is noteworthy to mention hereby, that herpes simplex encephalitis was reported following ChAdOx1 vaccine (108) however, this can be regarded as an infection-related AEFI rather than autoimmune induced.

In addition, a case report of a Japanese lady admitted who developed diplopia the next day following the first dose of the BNT162b2 vaccine administration was described in the literature (109). The symptoms aggravated after the second dose of the vaccine and the patients was finally diagnosed with encephalitis based on brain MRI findings. The patient responded well and totally recovered after treatment with steroids was initiated. The authors could not prove any causal relationship in their case.

In regard to the mRNA-1273 COVID vaccine, a case report of a patient diagnosed with acute encephalitis, myoclonus and Sweet syndrome was described after receiving the first dose of the mRNA-1273 vaccine (110). The symptoms resolved following glucocorticoids treatment.

### Myocarditis

Myocarditis is defined as the presence of inflammatory cellular infiltrate in the myocardium alongside tissue necrosis, which is not caused by coronary heart disease, and diagnosed by a combination of histological, immunological and immunohistochemical criteria (111). Based on etiologic factors, myocarditis can be divided into 2 subgroups: infectious, due to bacterial, viral, fungal, or parasitic infections; or non-infectious causes like autoimmunity, drugs, or vaccines (112). However, viral infections are seemingly the most common cause of myocarditis (113). Myocarditis can present with a range of symptoms from non-specific complaints such as fever and mild dyspnea, to fulminant hemodynamic imbalances and sudden death (114). In fact, myocarditis is considered as a common cause of sudden cardiac death (115). In a review article which included 1230 patients who initially had unexplained cardiomyopathy, 9% of the patients were eventually diagnosed with myocarditis (116). A link between vaccines and myocarditis was repeatedly reported in medical literature. For instance, in a review data of 35,188 individuals from the Vaccine Adverse Event Reporting System (VAERS), 8 cases of myocarditis were registered in individuals below 18 years of age, and 12 cases in elderly persons (117). However, it should be mentioned that VAERS presents some limitations, including the fact that it is a passive reporting system, and, therefore, information collected could be inaccurate, incomplete, coincidental, or unverifiable, warranting thorough epidemiological surveys to confirm such findings.

In another study designed to determine the incidence of cardiac symptoms and subclinical myocarditis/pericarditis after smallpox and trivalent influenza vaccine, out of 1081 individuals who received the smallpox vaccine, 4 Caucasian males were diagnosed with probable myocarditis and 1 female with suspected pericarditis. The study did not find any possible or probable cases of myocarditis/pericarditis following trivalent influenza vaccine however, some patients developed new cardiac symptoms like chest pain, dyspnea, and palpitations (118). One study that focused on myopericarditis following smallpox virus vaccination in the US military found that the incidence of myopericarditis was 7.5 times higher in soldiers who received the vaccine compared to the expected rate (119). Influenza vaccines as well were associated with cases of myocarditis (120–122). While myocarditis is well documented in regard to COVID-19 (123), the correlation with myocarditis and various types of COVID-19 vaccines is illustrated hereby.

#### BNT162b2 (BioNTech/Pfizer) and Myocarditis

Myocarditis was documented by several case reports in individuals after receiving the COVID-19 vaccines (124–126). Montgomery et al. (127) investigated the association between myocarditis and the mRNA COVID-19 vaccines in healthy military members of the US army. After approximately 2.8 million mRNA vaccine doses, 23 persons were diagnosed with myocarditis within 4 days of vaccination. Seven out of the 23 cases (30%) received the BNT162b2 vaccine and most developed the symptoms following the second dose. The study concluded that further evaluation of this rare adverse effect is warranted. Furthermore, several large-scale studies were conducted to evaluate this association in Israel. For instance, Barda and colleagues (128) evaluated 884,828 people vaccinated with BNT162b2, based on data from the largest health care organization in Israel. While the BNT162b2 vaccine was not associated with most of the side effects searched, the vaccine was strongly associated with increased risk of myocarditis. The risk was calculated as 2.7 events per 100,000 people, with highest risk among young men with a median age of 25. Another study conducted by Witberg et al. (129) searched specifically regarding the diagnosis of myocarditis among individuals in the largest health care organization in Israel that received at least one dose of the BNT162b2 vaccine. Among more than 2.5 million vaccinated members who were 16 years of age or older, the estimated incidence of myocarditis was 2.13 cases per 100,000 people. The highest incidence was documented in young male patients aged 16-29, the majority with mild to moderate disease. In addition, Mevorach and colleagues (130) followed the diagnosis of myocarditis in Israel after approximately 5.1 million people were vaccinated with two doses of the mRNA COVID-19 vaccines. A total of 283 persons developed symptoms of myocarditis, 142 (50%) occurred after the BNT162b2 vaccine, whereas 136 were eventually diagnosed with probable or definitive myocarditis. Most of the cases were mild and the highest incidence was recorded following the second dose and in young male patients aged 16-19 years. In comparison with unvaccinated people, the rate ratio 30 days after the second dose was 2.35 (95% CI, 1.10 to 5.02). The authors concluded that despite the low incidence, it increased after the BNT162b2 vaccine, all were mild in severity. Following the extension of the FDA authorization of the emergency use of the BNT162b2 vaccine to include children aged 12-16 in May 2021, Dionne et al. (131) presented a case series of 15 children with myocarditis after receiving the BNT162b2 vaccine. Similarly, myocarditis developed mainly in boys and after the second dose, all with a mild course.

#### mRNA-1273 (Moderna) and Myocarditis

Myocarditis is a rare post-mRNA-vaccine sequela, and was seen mostly among young, vaccinated males. Up to June 2021, around 1226 reports of myocarditis have been reported in the US after more than 296 million doses of mRNA COVID-19 vaccines administered (132). Symptoms usually began 3 days after vaccination with more than 75% of the cases happened after receiving the second dose of mRNA vaccine. The median age was 26 years of age with at least 56% of the affected people being younger than 30 years old. About 76% of the cases were found in men. Experts estimated the rate of post-vaccination myocarditis among young males aged 12-29 as 40.6 cases per million second doses of mRNA COVID-19 vaccines and 2.4 per million second doses among males aged > 30. On the other hand, a million vaccinations among young males aged 12-29 could prevent 560 hospitalizations, 138 intensive care unit (ICU) admissions, and six deaths associated with the COVID-19 infection. In turn, according to the UK MHRA, toward the end of November 2021, a total of 103 reports of myocarditis after the use of mRNA-1273 were registered. A rate of 37 suspected myocarditis cases per million doses were calculated for the mRNA-1273 COVID-19 vaccine (133). No death cases were registered. Additionally, in a study conducted by Diaz et al. (134) the mean monthly number of the cases of myocarditis during the vaccine period was found significantly higher than that before the vaccine was available (27.3 vs.16.9). The study included about 2 million subjects who received at least one dose of COVID-19 vaccine. Despite the fact that more people received the BNT162b2 vaccine than the mRNA-1273 vaccine (52.6% vs 44.4%), 55% of the cases occurred after receiving mRNA-1273 (11/20). Cases were mild, few required hospital admission, and discharged after a median of 2 days. No deaths or readmissions were reported. As mentioned earlier regarding 2.8 million doses of mRNA COVID-19 vaccines in healthy members of the US army, 23 cases of myocarditis were reported, 16 cases among persons who received the mRNA-1273 COVID-19 vaccine (127). The majority occurred after the second dose. In terms of possible pathogenetic explanation of the appearance of myocarditis, Bozkurt and colleagues (135) suggested the involvement of various mechanisms such as molecular mimicry between the viral spike protein and self-antigens. In addition, triggering of pre-existing dysregulated immune pathways in certain individuals, activation of immunologic pathways and dysregulated cytokine release, were also proposed. Regarding the higher rates of myocarditis among males, the difference in the immune response of sex hormones and underdiagnosed cases of myocarditis among women were suggested.

#### ChAdOx1 (AstraZeneca) and Myocarditis

While myocarditis has been extensively reported following mRNA COVID-19 vaccines, very few reports exist regarding the development of myocarditis in relation to the ChAdOx1 vaccine (136). In the latter, the patient was given a diagnosis of myopericarditis with pleuritis. The European Medicines Agency (EMA) estimated that 38 cases of myocarditis and 47 cases of pericarditis were attributed to ChAdOx1 vaccine out of 40 million vaccine doses administered in Europe until May 2021 (137). The agency ensured a monthly review of new cases but refuted the need for an accelerated investigation. In turn, the UK Health Security Agency dismissed a connection between the vaccine and reported cases, claiming that considering the extensive distribution of the ChAdOx1 vaccine in the UK, the cases are more likely attributed to the background incidence rate (138).

### Vaccine-Induced Immune Thrombotic Thrombocytopenia/Vaccine-Induced Prothrombotic Immune Thrombotic Thrombocytopenia

Since the appearance of the vaccine-induced immune thrombotic thrombocytopenia (VITT)/vaccine-induced prothrombotic immune thrombotic thrombocytopenia (VIPITT) was mainly attributable to the adenoviral vector vaccines, the ChAdOx1 (AstraZeneca) and the Ad26.COV2.S (Janssen/Johnson & Johnson) vaccines, the current section starts with the AstraZeneca vaccine, then the mRNA vaccines follow.

#### ChAdOx1 (AstraZeneca) and VITT/VIPITT

**Figure 1:**
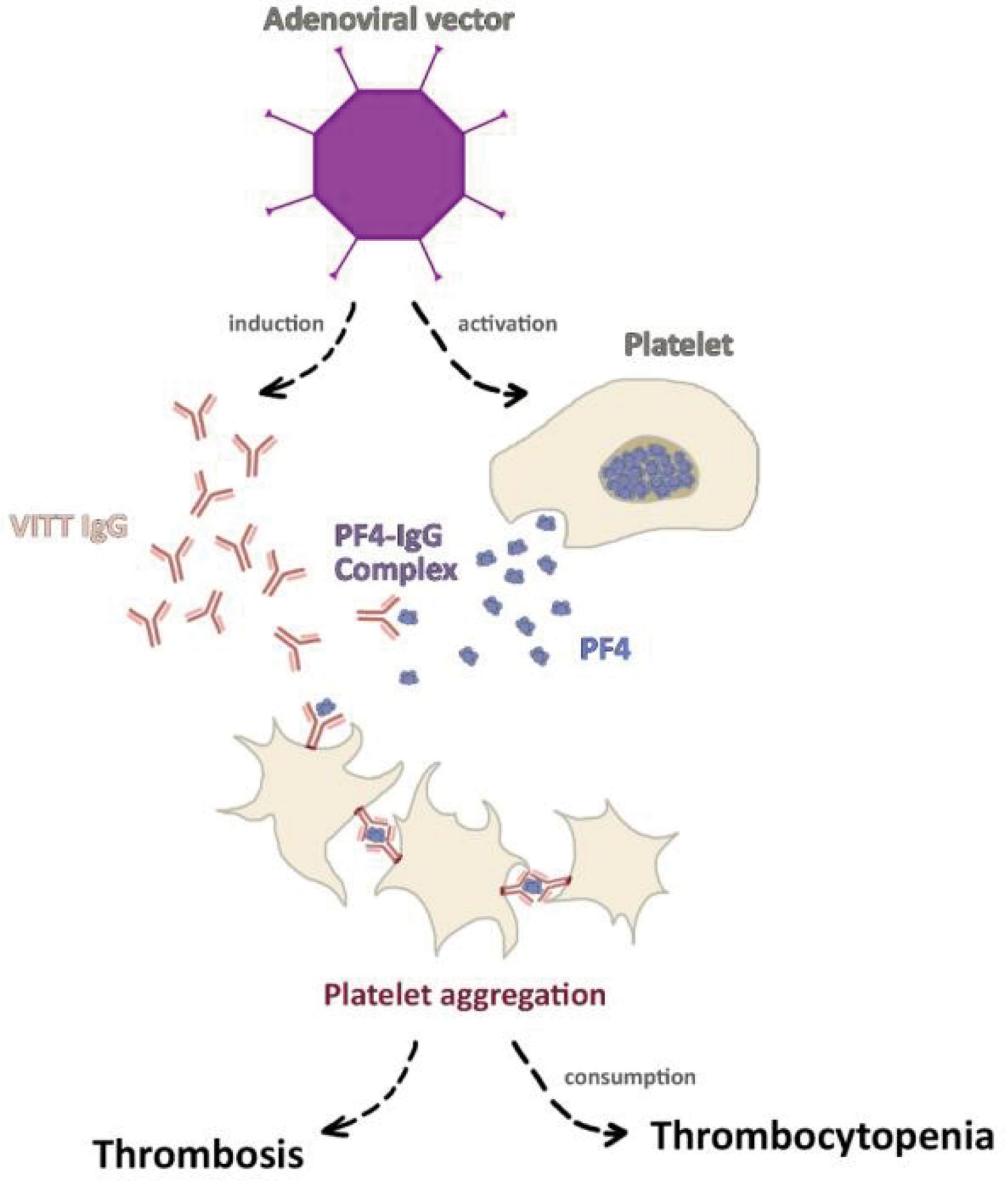
The pathogenesis of vaccine-induced immune thrombotic thrombocytopenia (VITT). Vector-based COVID-19 vaccines, particularly ChAdOx1 COVID-19 vaccine was shown to induce platelet activation alongside platelet factor 4 (PF4) autoantibodies formation. The activated platelets secret greater amount of PF4 which binds to autoantibodies forming the PF4-IgG complex. As a result, FcγRIIA receptor mediated platelet activation leads to widespread thrombosis and secondary thrombocytopenia.

In early 2020, as the newly authorized COVID-19 vaccinations were being distributed worldwide, cases of thrombotic events as well as thrombocytopenia following vaccination with the ChAdOx1 vaccine began to emerge (139–141). While the EMA and the WHO issued statements declaring the reported incidence was not enough to deduce causation and cautioning against premature pausing of vaccination programs (142, 143), numerous European countries decided to halt the use of the vaccine pending further investigations. Subsequently, larger studies demonstrated slightly increased rates of venous thromboembolic events among the adenoviral vector vaccine recipients (144, 145). A thorough investigation by the Pharmacovigilance Risk Assessment Committee (PRAC) introduced for the first time the notion of VITT/VIPITT (146), as a rare adverse reaction to the vaccine. VITT/VIPITT is defined as presence of venous or arterial thrombosis, thrombocytopenia, and autoantibodies (anti-PF4–polyanion or anti-PF4–heparin antibodies) within 5–30 days of vaccination with either AstraZeneca or Janssen/Johnson & Johnson COVID-19 vaccines (147). In fact, VITT/VIPITT shares many similarities with heparin-induced thrombocytopenia (HIT) as both disorders are facilitated by platelet factor 4 (PF4) autoantibodies leading to platelet activation and consumption (147). The trigger for the autoantibody formation is poorly understood, as the cases reported had no previous exposure to heparin (148), and the autoantibodies were not found to cross-react with the viral spike proteins indicating a previous infection (139). Furthermore, epidemiological data illustrated that over 85% of VITT/VIPITT cases occurred in women under 60 years of age, despite higher rates of vaccination among the elderly (146). Therefore, the findings supported the assumption that VITT/VIPITT is most likely to be an autoimmune phenomenon. While its exact pathogenesis has not been established yet, a recent study demonstrated that ChAdOx1 COVID-19 vaccine induces higher rates of inflammation and platelet activation compared to other COVID-19 vaccines (149). The VITT/VIPITT autoantibodies consequently bind to PF4 (150), a chemokine secreted from activated platelets, in a site that corresponds to the heparin-binding site. In turn, immune complexes formation induces FcγRIIA receptor mediated platelet activation leading to widespread thrombosis with secondary thrombocytopenia due to platelet consumption (**Figure 1**) (148, 150). As the ChAdOx1 vaccination has proven to be effective in prevention of COVID-19 infection (151, 152), and due to the rarity of serious adverse events such as VITT/VIPITT, it is important to view this phenomenon in the proper clinical context.

#### BNT162b2 (BioNTech/Pfizer) and VITT/VIPITT

Since the mass vaccination campaign began with the BNT162b2 vaccine, several case reports have been published regarding the combination of thrombotic events together with thrombocytopenia, in a form of cerebral venous sinus thrombosis, VITT/VIPITT, and acquired thrombotic thrombocytopenic purpura (TTP) several days following vaccination with the BNT162b2 vaccine (153–162). In a large-scale study based on national data made up of 29,121,633 vaccinated individuals with the first dose of BNT162b2 vaccine (163), the association between VITT/VIPITT occurring as post-vaccine and post-infection was investigated. Among the enrollees, 9,513,625 were vaccinated with the BNT162b2 vaccine, and 1,758,095 were in the post-infection group as they were previously infected with COVID-19. An increased risk of arterial thromboembolism, cerebral venous sinus thrombosis, and ischemic stroke after the BNT162b2 vaccine was found however, an even higher risk of these associations was documented following COVID-19 infection. The study concluded an increased risk of hematological and vascular events for short-time intervals following the BNT162b2 vaccine. Still, most of these events were substantially higher and more prolonged after COVID-19 infection than after vaccination in the same population. In contrast, in another large-scale study using a national prospective cohort in Scotland with regard to hematologic and vascular events following vaccination with various vaccine types, no positive correlations were found between BNT162b2 and thrombocytopenic, thromboembolic, and hemorrhagic events (164).

#### mRNA-1273 (Moderna) and VITT7VIPITT

Cases of ITP and TTP after mRNA-1273 COVID-19 vaccine have been documented since the early vaccination campaign (165–168). A flare of familial thrombocytopenia has been reported as well (169). In addition, 13 cases of thrombocytopenia following 16 million doses of mRNA-1273 were found in the USA, suggesting a rate of 0.8 cases per million (170). Lee et al. (171) discussed 20 cases of thrombocytopenia among 20 million people who were vaccinated by at least one dose of the BNT162b2 or the mRNA1273 vaccines. A total of 17 cases considered new-onset ITP, and 11 out of the 20 cases received mRNA-1273. Furthermore, in a study based on the WHO Vigibase which includes 361 million COVID-19 vaccinated people, 2161 thrombotic events were noted, 325 of the cases appeared after the mRNA-1273 vaccine (172). Among the 325 thrombotic events, only 8 had associated thrombocytopenia. Regarding VITT/VIPITT, a German study assessed the rate of cerebral sinus and venous thrombosis (CVT) within 1 month of the first dose of the BNT162b2, ChAdOx1 and mRNA‐1273 COVID-19 vaccines and the frequency of VITT/VIPITT as the causing mechanism (173). The authors identified 45 cases of CVT, none of the cases occurred after the administration of mRNA-1273. Similar findings were shown by Krzywicka et al. (174) as out of 213 European patients with CVT, only one patient received the mRNA-1273 vaccine. According to the same study, out of 107 patients who had CVT alongside thrombocytopenia, no patient received the mRNA-1273 vaccine. In addition, only 5 possible cases of CVT were reported in Europe among 4 million subjects who received the mRNA-1273 vaccine (175). In summary, the risk of thrombocytopenia and thrombotic events in mRNA-1273 vaccinated people appears to be very low. In fact, the incidence of CVT among people vaccinated with the mRNA COVID-19 vaccines, both the BNT162b2 and mRNA-1273, was shown to be lower than that among people infected with COVID-19 (4.1 vs 39 per million) (176).

### Other Autoimmune Side Effects

#### Immune Thrombocytopenia

ITP is a well-known autoimmune hematological condition characterized by a substantial reduction in peripheral platelet count to less than <100,000/microL due to platelet destruction by antiplatelet antibodies (177). Patients generally asymptomatic, may have minor mucocutaneous bleeding, and not uncommonly progress to life-threatening hemorrhages in severe cases (178). ITP was reported in correlation to COVID-19, COVID-19 vaccines, as well as other vaccines (179, 180).

#### Minimal Change Disease

Minimal change disease (MCD) is a histologically based renal pathology which is the leading cause of idiopathic nephrotic syndrome in adults and children (181). As cases of MCD were described after vaccination in the past (182–184), the reporting of cases following COVID-19 vaccines were not unforeseen.

##### BNT162b2 (BioNTech/Pfizer) and MCD

Several case reports have been published regarding new-onset or relapse of MCD following vaccination with the BNT162b2 vaccine. A 50-year-old healthy male presented with nephrotic syndrome and acute kidney injury four days following the first dose of the BNT162b2 vaccine (185). The diagnosis was confirmed by kidney biopsy. Renal function returned to normal within few days following treatment with corticosteroids. Following this report, similar cases in older people were presented (186, 187). The symptoms appeared seven days following the first dose of the vaccine. MCD was confirmed by kidney biopsy as well. In addition, a relapse of MCD was also described in a patient diagnosed with MCD 20 years prior to vaccination, developed proteinuria after vaccination, which resolved within 2 weeks following corticosteroids and cyclosporine treatment (188).

##### mRNA-1273 (Moderna) and MCD

Though autoimmune renal AEFIs of the mRNA-1273 COVID-19 vaccine were reported, MCD was not common among them. For instance, Thappy et al. (189) presented a case of 43-year-old man who developed symptoms of minimal change disease 7 days after receiving the first dose of the mRNA-1273 vaccine. The biopsy confirmed concomitant MCD and IgA nephropathy. The patient responded well to oral steroid treatment. In addition, biopsy proven IgA nephropathy, both as new onset and flare, was previously described following the mRNA-1273 COVID-19 vaccine, manifesting as hematuria (190, 191). Interestingly, the symptoms appeared 1-2 days after the second dose of the vaccine.

##### ChAdOx1 (AstraZeneca) and MCD

Several cases of MCD were described in the context of the ChAdOx1 vaccine (192–194). All these cases presented with a clinical picture of nephrotic syndrome that started up to 15 days after the first dose of the vaccine. The fairly short period from vaccination to presentation could be attributed to cytokine release by activated T-cells, as the ChAdOx1 vaccine has been shown to induce a robust T-cell response in most individuals (195). However, as the reported cases were limited, a definite conclusion could not be drawn. The number of cases of MCD following the vaccine was small, thus a strong association could not be inferred.

#### Vasculitis

As for vasculitis, two cases of antineutrophil cytoplasmic antibody (ANCA) associated vasculitis after the second dose of the mRNA-1273 COVID-19 vaccine were reported. As a result of the new onset ANCA vasculitis, one patient became dependent on dialysis (196), while in the other case renal improvement was achieved after plasma exchange, pulse steroid and cyclophosphamide therapy (197). Additional case of ANCA vasculitis which manifested as renal failure together with pulmonary hemorrhage 3 weeks after the first dose of mRNA-1273 was also described (198).

#### Miscellaneous

Autoimmune hepatitis was reported as well among individuals vaccinated with various types of COVID-19 vaccines (199–202). In a multicenter study conducted in several countries during the early implementation of the program of vaccination against the COVID-19, Watad and colleagues (203) investigated the association between new-onset or flares of immune-mediated diseases 28-days following mRNA COVID-19 vaccination. The authors detected 27 cases; 17 (63%) had flares of their illnesses whereas 10 (37%) cases had a new-onset disease. In total, 23 (85%) patients received the BNT162b2 vaccine, 2 (7.5%) received the mRNA-1273 vaccine, and 2 (7.5%) received the ChAdOx1 vaccine. Taking into consideration the great proportion of people vaccinated, the authors concluded that immune-mediated diseases flares or new-onset temporally associated with COVID-19 vaccination are rare. Furthermore, Ishay et al. (204) presented a case series of patients with new-onset or flares of autoimmune AEFIs after the BNT162b2 vaccine. Eight patients presented with either symmetric polyarthritis, panuveitis, pericarditis, temporal arteritis-like disease, fever of unknown origin (FUO), oligoarthritis, and myocarditis following either the first dose (62.5%) or the second dose (37.5%) of the vaccine. Based on their findings, the authors concluded that immune AEFIs might occur following vaccination however, they usually follow a mild course.

A brief summary of the studies addressing autoimmune AEFIs of the most utilized COVID-19 vaccines, including the study population and the conclusion of the works, is presented in **Table 1**.

**Table 1:** A brief summary of studies addressing immune and autoimmune side effects of COVID-19 vaccines.

Side effect	Vaccine	Authors	Study population	Conclusions
**Neurological Autoimmunity**	Pfizer (BNT162b2)	Trimboli et al. (205)	1 case of GBS after receiving the second dose of BNT162b2 COVID-19 vaccine	Authors believe that the clinical and laboratory findings including the lack of overt trigger are consistent with a causal association between GBS and Pfizer^®^ anti-SARS-CoV-2 vaccine
		Bouattour et al. (50)	1 case of GBS after 1^st^ dose of BNT162b2 vaccine	A patient who developed GBS 7 days after receiving the first dose of Pfizer-BioNTech COVID-19 vaccine
		Shapiro Ben David et al. (52)	Retrospective cohort study in the second largest health maintenance organization in Israel, diagnosis code for GBS after receiving at least 1 vaccine dose	In this cohort study, which included 702 patients, only 1 needed short medical care for relapse of previous syndrome, which represents a minimal risk.
		Garcia-Grimshaw et al. (54)	Analysis of cohort of 3,890,250 Hispanic/Latinx recipients of the BNT162b2 mRNA vaccine (613,780 of for incident GBS occurring within 30 days from vaccination	Among recipients of the BNT162b2 mRNA vaccine, GBS may occur at the expected community-based rate
		Shasha et al. (53)	Individuals ≥16 years vaccinated with at least one dose of BNT162b2	No association was found between vaccination, Bell’s palsy, herpes zoster or GBS. This study adds reassuring data regarding the safety of the BNT162b2 vaccine.
		Garcia-Grimshaw et al. (76)	Prospective observational cohort of 700,000 patients from a database of all systemic and neurologic adverse events following immunization	Non-serious events occurred in less than 1% of recipients, while serious ones occurred in only 33 (0.005%) recipients, suggesting that the vaccine is not only effective but also safe
		Waheed et al. (49)	1 case of GBS presenting after 1^st^ vaccine	An 82 y/o female who presented 2 weeks after vaccination with difficulty in walking
		Nishiguchi et al. (22)	1 case of MFS presenting after 1^st^ vaccine	Authors report the first case of COVID-19 vaccination-associated MFS. However, it is difficult to deny that this result may be a coincidence in time, and therefore, no cause-and-effect relationship can be concluded at this time.
		Razok et al. (51)	1 case of GBS presenting after 2^nd^ vaccine	The patient presentd with acute flaccid paralysis (AFP) after receiving the COVID-19 vaccine.
		Miyaue et al. (75)	1 case of LETM presenting after 1^st^ vaccine	This case meets the criteria for a “probable” AEFI, considering the following features: (i) the onset of neurological symptoms occurred within a week after vaccination; (ii) the patient had no previous neurological symptoms after other vaccines, nor symptoms suggestive of prior infection; and (iii) no other cause of LETM was identified on a thorough diagnostic evaluation.
		Colella et al. (96)	1 case of Bell’s palsy after 1^st^ vaccine	Although a causal relationship cannot be established for most rare adverse events, the timing and mode of onset of the palsy strongly suggests that it was related to BNT162b2 vaccine injection.
		Shibli et al. (98)	Population based study in Israel comparing expected cases of Bell’s palsy with number of cases after 1^st^ and 2^nd^ vaccine dose	The overall observed rate of Bell’s palsy after vaccination was higher than the expected rates
		Kobayashi et al (109)	1 case of encephalitis after 1^st^ dose, exacerbated after 2^nd^ dose.	No evidence of causal relationship was found.
	Moderna (mRNA-1273)	Masuccio et al. (56)	1 case of GBS after 2^nd^ vaccine	According to clinical features, a subacute GBS might be reasonably hypothesized after the administration of COVID-19 mRNA-1273 vaccine second dose, with about 6 weeks elapsing between the vaccination and the symptoms onset.
		Kania et al. (77)	1 case of acute disseminated encephalomyelitis after 1^st^ vaccination	The patient manifested a typical radiological pattern for ADEM with extensive, diffuse demyelinating lesions in the brain and along all cervical and thoracic spinal cord
		Fujikawa et al. (78)	1 case of neuromyelitis optica spectrum disorder presenting after 1^st^ vaccination	Considering the temporal association between administration of the vaccine, onset of patient’s symptoms, and previous reports of post-vaccination NMOSD, patient’s NMOSD was triggered by the SARS-CoV-2 mRNA-1273 vaccine.
		Gao et al. (79)	1 case of LETM after 1^st^ vaccination	The temporal relationship between vaccination and ATM in the case was clinically reasonable (48 h post-vaccination)
		Cellina et al. (99)	1 case of Bell’s palsy after 1^st^ vaccination	The patient complained of symptoms at 12 h from the injection. The timing of Bell’s palsy onset after mRNA vaccine administration varies
		Iftikhar et al. (100)	1 case of Bell’s palsy after 2^nd^ vaccination	This case highlights the importance of vaccine history in patients presenting to the emergency department with Bell’s palsy. COVID-19 mRNA vaccines can be considered as an additional possible risk factor in the etiology of Bell’s palsy.
		Martin-Villares et al. (101)	1 case of Bell’s palsy after 1^st^ vaccination	Evidence of a temporal association between the vaccine administration and the facial nerve palsy is clear: Bell´s palsy appeared 2 days after the administration of the mRNA COVID-19 vaccine
		Torrealba-Acosta et al. (110)	1 case of encephalitis and Sweet syndrome after 1^st^ dose	Though temporal relation was described, causality could not be proven
	AstraZeneca (ChAdOx1)	Min et al. (66)	2 case presentations + review of 12 published cases	The two patients shared many clinical features: pure sensory manifestations, short-latency from vaccination to onset, progression duration, and no serum antibodies against gangliosides. Sensory GBS was considered the most probable diagnosis.
		Oo et al. (67)	4 case presentations + review of 15 published cases	These four cases can lend further weight to the likely causal link between COVID-19 vaccine AZ and GBS
		McKean et al. (65)	1 case of GBS following the 1st vaccination	This is the first reported case of GBS which was temporally related to the Vaxzevria vaccine in Malta.
		Introna et al. (62)	1 case of GBS following the 1st vaccination	A case of GBS presenting with papilledema as atypical onset
		Allen et al. (59)	4 cases of GBS following 1st vaccination	There was an interval of 11 to 22 days between vaccination and symptom onset.
		Notghi et al. (86)	1 case of LETM after 1st vaccination	58-year-old man admitted to hospital 10 days after his first AstraZeneca COVID-19 vaccination with progressive neurological symptoms and signs, and investigations and imaging consistent with LETM
		Pagenkopf et al. (87)	1 case of LETM after 1st vaccination	The case of LETM presented here shows a close temporal association to COVID-19 vaccination, as symptoms occurred within 11 days post injection of first dose AZD1222, AstraZeneca
		Helmchen et al. (206)	1 case of LETM in a patient with MS after 1st vaccination	The case suggests that the vector-based COVID-19 vaccine should not be used in RRMS if mRNA vaccines are available.
		Voysey et al. (81)	Evaluation of 4 controlled trials in 3 countries (11636 patients)	ChAdOx1 nCoV-19 has an acceptable safety profile and has been found to be efficacious against symptomatic COVID-19 in this interim analysis of ongoing clinical trials.
		Hsiao et al. (84)	1 case of acute transverse myelitis after 1st vaccination	Although they rarely occur, the association of the COVID-19 vaccine and the disease, along with other neurological complications, should not be ignored
		Malhotra et al. (85)	1 case of acute transverse myelitis after 1st vaccination	Considering an incidence of 1–4 cases per million per year, 6 an event of myelitis occurring after more than 50 million vaccine doses appears fairly acceptable
		Bonifacio et al. (60)	5 cases of bilateral facial weakness after 1^st^ vaccination	The incidence of five cases of the very uncommon BFP variant of GBS occurring within 2 weeks of Vaxzevria is further suggestive of an etiological link.
		Hasan et al. (61)	1 case of paresthesia and progressive weakness presenting after 1^st^ case	No direct link could be ascertained
		Kanabar et al. (63)	2 patients with GBS presenting after 1^st^ vaccination	Both patients described in this report had bilateral facial weakness at presentation
		Maramattom et al. (64)	7 patients who presented with GBS after 1^st^ vaccination	Patients were in their 5^th^ to 7^th^ decades of life and predominantly female. All patients progressed to areflexic quadriplegia, and six of the seven cases required mechanical ventilation for respiratory failure. All seven cases had bilateral facial paresis, which usually occurs in fewer than 20% of unselected GBS cases
		Tan et al. (88)	1 case of LETM after 1^st^ vaccination	Although TM following vaccination is rare, the temporal causality of LETM, in this case, is undeniable
		Zuhorn et al. (107)	3 cases of encephalitis, one after the 1^st^ dose, others not mentioned	The complication of autoimmune encephalitis after ChAdOx1 nCoV-19 vaccination appears to be very rare. Clearly, the benefit of vaccination outweigh the risks
	Multiple vaccine types	Kaulen et al. (207)	21 consecutive cases of neurological autoimmunity, which occurred 3–23 days following SARS‐CoV‐2 vaccinations	A large series of neurological autoimmunity in temporal association with various SARS‐CoV‐2 vaccines (BNT162b2, ChAdOx1 and mRNA‐1273) is reported
		Koh et al. (103)	A prospective study at 7 acute hospitals in Singapore of hospitalized patients who were referred for neurological complaints and had COVID-19 mRNA vaccines	Over a 4-month period during which approximately 1.4 million people received the COVID-19 mRNA vaccines, authors recorded a spectrum of neurological disorders in only 457 hospitalized patients
		Loo et al. (57)	A retrospective study examining all persons presenting with acute-onset polyradiculoneuropathy from January 1, 2021, to June 30, 2021 who were admitted to UK hospitals	Most cases identified in the study (87.5%) occurred after the AstraZeneca vaccine
		Renoud et al. (104)	133,883 cases of adverse drug reactions reported with mRNA COVID-19 vaccines in the World Health Organization pharmacovigilance database	When compared with other viral vaccines, mRNA COVID-19 vaccines did not display a signal of facial paralysis
		Ismail et al. (80)	Review of 32 cases of CNS demyelination after all vaccine types	CNS demyelination was reported following all types of authorized COVID-19 vaccines (no protein-based vaccine was authorized at the time of writing). Neurological symptoms appeared within the first 1–2 weeks in most cases. Females comprised the majority of cases. Furthermore, more than half of the cases had history of probable or definite autoimmune diseases
		Ozonoff et al. (95)	Literature review of Bell’s palsy after all types of COVID vaccines	The observed incidence of Bell’s palsy after mRNA vaccines is between 3·5-times and 7-times higher than would be expected in the general population.
		Sato et al. (102)	Analysis of Bell’s palsy cases databases after mRNA vaccines	The incidence of facial nerve palsy as a non-serious AEFI may be lower than, or equivalent to, that for influenza vaccines.
		Patone et al. (106)	Case series studies investigating hospital admissions from neurological complications after 1^st^ dose of AstraZeneca or Pfizer vaccines	Authors found an increased risk of hospital admission for GBS (15–21 days and 22–28 days), Bell’s palsy (15–21 days) and myasthenic disorders (15–21 days) in those who received the ChAdOx1nCoV-19 vaccine. Second, an increased risk of hospital admission for hemorrhagic stroke (1–7 days and 15–21 days) was observed in those who received the BNT162b2 vaccine
**Myocarditis**	Pfizer (BNT162b2)	Barda et al. (128)	884,828 people on a nation-wide setting	The vaccine was associated with an excess risk of myocarditis (1 to 5 events per 100,000 persons)
		Montgomery et al. (127)	23 male patients within the US Military Health System who experienced myocarditis after COVID-19 vaccination between January and April 2021.	The consistent pattern of clinical presentation, rapid recovery, and absence of evidence of other causes support the diagnosis of hypersensitivity myocarditis
		Abu Mouch et al. (124)	6 cases of myocarditis, which occurred shortly after BNT162b2 vaccination	Five patients presented shortly after the second vaccine dose and one patient presented 16 days after receiving his first vaccine dose
		Mevorach et al. (130)	Retrospectively review data obtained from December 20, 2020, to May 31, 2021, regarding all cases of myocarditis in Israel	The incidence of myocarditis, although low, increased after the receipt of the BNT162b2 vaccine, particularly after the second dose among young male recipients. The clinical presentation of myocarditis after vaccination was usually mild.
		Larson et al. (125)	8 patients 2-4 days post mRNA-based vaccine	The temporal association between receiving an mRNA-based COVID-19 vaccine and the development of myocarditis is notable, potentially supporting the hypothesis that myocarditis could be an mRNA vaccine–related adverse reaction
		Dionne et al. (131)	Case series of children younger than 19 years hospitalized with myocarditis within 30 days of BNT162b2 vaccine.	Myocarditis was diagnosed in children after COVID-19 vaccination, most commonly in boys after the second dose
		Witberg et al. (129)	Nationwide Israeli cohort through Health care service database evaluating myocarditis cases after Pfizer vaccines	The estimated incidence of myocarditis was 2.13 cases per 100,000 persons; the highest incidence was among male patients between the ages of 16 and 29 years. Most cases of myocarditis were mild or moderate in severity.
	Moderna (mRNA-1273)	Gargano et al. (132)	296 million doses of administered mRNA (Pfizer and Moderna) COVID-19 vaccines up to June 11,2021 in the US.	Myocarditis reporting rates were 40.6 cases per million second doses of mRNA COVID-19 vaccines administered to males aged 12−29 years and 2.4 per million second doses administered to males aged ≥30 years
		UK Medicines & Healthcare products Regulatory Agency (133)	1.5 million recipients of the first doses and approximately 1.3 million recipients of the second doses of mRNA-1273 in the UK.	The expected benefits of the vaccines in preventing COVID-19 and serious complications associated with COVID-19 far outweigh any currently known side effects in the majority of patients.
	AstraZeneca (ChAdOx1)	Hung et al. (136)	1 case of myopericarditis with pleuritis	Symptoms occurred 7 days post-vaccination, and the patient was hospitalized for 12 days with a total recovery. Due to a negative result for other etiologies, the possibility of vaccine-related myopericarditis with bilateral pleural effusion cannot be totally excluded.
	Multiple vaccine types	Vidula et al. (126)	Two healthy young patients with clinically suspected myocarditis after receiving an mRNA-based COVID-19 vaccine	While endomyocardial biopsy was not performed, both patients met the diagnostic criteria for clinically suspected myocarditis. The temporal association of the receipt of the vaccine and absence of other plausible causes suggest the vaccine as the likely precipitant
		Diaz et al. (134)	2,000,287 individuals receiving at least 1 COVID-19 vaccination in the US	This study shows that myocarditis after vaccination is primary seen in younger male individuals a few days after the second vaccination. Pericarditis may be more common than myocarditis among older patients.
**Vaccine-induced immune thrombotic thrombocytopenia (VITT) and other coagulation abnormalities**	Pfizer (BNT162b2)	Maayan et al. (158)	4 patients from two academic medical centers who developed TTP were identified from mid- February to mid- March 2021	A disintegrin and metalloproteinase with a thrombospondin type 1 motif, member 13 (ADAMTS13) activity should be evaluated in patients with history of aTTP before and after any vaccination, especially the SARS‐CoV‐2 vaccination
		de Bruijn et al. (154)	1 case report of TTP after first BNT162b2 vaccine	This is the first case report of iTTP after mRNA-based COVID-19 vaccination in a previously TTP-naïve patient.
		Akiyama et al. (153)	1 case of ITP after 1^st^ vaccination	An extremely rare case of secondary ITP presumed to have occurred after BNT162b2 vaccination
		Dias et al. (155)	2 cases of thromboembolism after 1^st^ vaccination	In both patients, there was no evidence of thrombocytopenia or antiplatelet antibodies, and alternative causes for cerebral venous thrombosis were found. As such, despite the temporal relation of both cases to vaccine administration, these types of cerebral venous thrombosis do not seem to be pathophysiological different from cerebral venous thrombosis not associated to SARS-CoV-2 vaccination
		Ganzel et al. (156)	1 case of ITP after 1^st^ vaccination	May have a temporal relationship with administration of the Pfizer-BioNTech COVID-19 vaccine
		King et al. (157)	1 case of ITP after 2^nd^ vaccination	ITP should be considered a severe AE of the BNT162b2 mRNA COVID-19 vaccine.
		Matsumura et al. (159)	2 cases of ITP after 1^st^ vaccination	Whether or not ITP is triggered by the vaccination or not is difficult to identify
		Rodríguez et al. (160)	1 case of VITT after 1^st^ vaccination	This case meets the Brighton Collaboration case definition of VITT, with thrombocytopenia and thrombosis without prior heparin exposure
		Waqar et al. (161)	1 case of TTP after 2^nd^ dose of vaccination	Further studies are, however, needed to verify possible associations between microangiopathic, thrombocytopenic thrombotic disorders and the administration of vaccines against COVID-19
		Yoshida et al. (162)	1 case of TTP after 1^st^ vaccination	The first case of acquired TTP in Japan may have been associated with the first dose of the BNT162b2 mRNA COVID-19 vaccine
	Moderna (mRNA-1273)	Hines et al. (165)	1 case of ITP after 1^st^ vaccination	The temporal relationship of her vaccination with thrombocytopenia and abnormal liver enzymes points towards the Moderna mRNA-1273 SARS-CoV-2 vaccine as the most likely inciting factor
		Karabulut et al. (166)	1 case of TTP in a patient with known ITP and TTP after 1^st^ vaccination	The close temporal association between vaccine administration, recent COVID-19, and relapse of remitted TTP raises concern for an enhanced immune reaction to COVID-19 vaccine in the setting of recent COVID-19 and underlying autoimmune disease
		Malayala et al. (167)	1 case of thrombocytopenia after 1^st^ vaccination	Authors attribute this thrombocytopenia and purpuric rash as the side effects of the mRNA-1273 vaccine
		Su et al. (168)	1 case of VITT in a patient with pancreatic cancer after 1^st^ vaccination	This case study was the first to report a cancer patient who was diagnosed with VITT after mRNA-1273 vaccination
		Toom et al. (169)	Patient with ITP who presented with flareup after 1^st^ vaccination	The temporal sequence of the events suggests an exacerbation of patient’s chronic thrombocytopenia related to the receipt of the mRNA‐1273 Covid‐19 vaccine
	AstraZeneca (ChAdOx1)	Greinacher et al. (139)	11 patients in Germany and Austria with thrombocytopenia and clotting	Vaccination with ChAdOx1 nCov-19 can result in the rare development of immune thrombotic thrombocytopenia mediated by platelet-activating antibodies against PF4, which clinically mimics autoimmune heparin-induced thrombocytopenia
		Schultz et al. (140)	5 patients in Norway (healthcare workers) with thrombocytopenia and clotting	Five cases occurred in a population of more than 130,000 vaccinated persons, they represent a rare vaccine-related variant of spontaneous heparin-induced thrombocytopenia
		Pottegård et al. (145)	282572 patients in Norway and Denmark who experienced clotting events after vaccination	Excess rate of venous thromboembolism, including cerebral venous thrombosis, among recipients of the Oxford-AstraZeneca covid-19 vaccine ChAdOx1-S within 28 days of the first dose
		Perry et al. (144)	99 patients from 43 hospitals in the UK with clotting and thrombocytopenia	Cerebral venous thrombosis appears to be more severe in the context of VITT
		Scully et al. (141)	23 patients who presented with thrombosis and thrombocytopenia after 1^st^ vaccination	Testing for antibodies to platelet factor 4 (PF4) was positive in 22 patients (with 1 equivocal result) and negative in 1 patient
	Multiple vaccine types	Schulz et al. (173)	45 CVT cases occurring after 7,126,434 first vaccine doses of all types- using official statistics of 9 German states.	The findings point toward a higher risk for CVT after ChAdOx1 vaccination, especially for women
		Krzywicka et al. (174)	213 European patients with CVT after any vaccination	Cerebral venous sinus thrombosis occurring after ChAdOx1 nCov-19 vaccination has a clinical profile distinct from CVST unrelated to vaccination. Only CVST after ChAdOx1 nCov-19 vaccination was associated with thrombocytopenia
		Welsh et al. (170)	Case-series study of thrombocytopenia after mRNA vaccines using Vaccine Adverse Event Reporting System (VAERS)	The number of thrombocytopenia cases reported to the Vaccine Adverse Event Reporting System (VAERS) does not suggest a safety concern attributable to mRNA COVID-19 vaccines at this time
		Cines et al. (175)	4 million subjects which received any vaccine type in Europe	Cases of immune thrombocytopenia and bleeding without thrombosis that were induced or revealed after exposure to the messenger RNA (mRNA)–based vaccines produced by Moderna (mRNA-1273) and Pfizer–BioNTech (BNT162b2). The study has now highlighted three independent descriptions of 39 persons with a newly described syndrome characterized by thrombosis and thrombocytopenia that developed 5 to 24 days after initial vaccination with ChAdOx1 nCoV-19 (AstraZeneca)
		Lee et al. (171)	20 million people who have received at least one dose of Pfizer or Moderna vaccines. in the USA	The possibility that the Pfizer and Moderna vaccines have the potential to trigger *de novo* ITP (including clinically undiagnosed cases) cannot be excluded, albeit very rarely. Distinguishing vaccine‐induced ITP from coincidental ITP presenting soon after vaccination is impossible at this time.
		Smadja et al. (172)	361 million vaccinated people from the whole world with any vaccine type	The authors suggest that thrombotic events, including CVT, might occur in association with all three vaccines, but this hypothesis requires further investigations
		Torjesen et al. (176)	Using a US electronic health records, comparing incidence of cerebral venous thrombosis in patients two weeks after a COVID-19 diagnosis with that in patients two weeks after COVDI-19 vaccination in all vaccine types	SARS-CoV-2 infection is associated with more risk for CVT than COVID-19 mRNA vaccines
		Hippisley-Cox et al. (163)	Using a UK national data on covid-19 vaccination (AstraZeneca or Pfizer) and hospital admissions due to thrombocytopenia, venous thromboembolism, and arterial thromboembolism	Increased risks of hematological and vascular events that led to hospital admission or death were observed for short time intervals after first doses of the ChAdOx1 nCoV-19 and BNT162b2 mRNA vaccines. The risks of most of these events were substantially higher and more prolonged after SARS-CoV-2 infection than after vaccination in the same population.
		Simpson et al. (164)	National prospective cohort estimating hematological and vascular adverse events after 1^st^ vaccination with either AstraZeneca or Pfizer	A first dose of ChAdOx1 was found to be associated with small increased risks of ITP, with suggestive evidence of an increased risk of arterial thromboembolic and hemorrhagic events
**Autoimmune hepatitis**	Pfizer (BNT162b2)	Avci et al. (199)	1 case report after 1^st^ BNT162b2 vaccine	Although the exact cause of autoimmune reactions is unknown, an abnormal immune response and bystander activation induced by molecular mimicry is considered a potential mechanism, especially in susceptible individuals
	Moderna (mRNA-1273)	Zin Tun et al. (202)	1 case report of autoimmune hepatitis with Moderna vaccine+ review of other cases	This case illustrates immune-mediated hepatitis secondary to the Moderna vaccine, which on inadvertent re-exposure led to worsening liver injury with deranged synthetic function
	AstraZeneca (ChAdOx1)	Rela et al. (18)	2 cases of AIH following 1^st^ vaccination	There were no clear clinical or biochemical features apart from a chronological association to differentiate patients’ vaccine-related AIH from idiopathic AIH.
		Clayton-Chubb et al. (200)	1 case of AIH following 1^st^ vaccination	This case supports the notion of COVID-19 vaccine-triggered autoimmune phenomena irrespective of the vaccine’s mechanism of action, though this is the first report of an adenovirus-based vaccine precipitating AIH
**Minimal Change Disease (MCD)**	Pfizer (BNT162b2)	Lebedev et al. (185)	1 case report after 1^st^ BNT162b2 vaccine	The association between the vaccination and MCD is at this time temporal and by exclusion, and by no means firmly established
		D’Agati et al. (186)	1 case report after 1^st^ BNT162b2 vaccine	The strong temporal association with vaccination suggests a rapid T cell–mediated immune response to viral mRNA as a possible trigger for podocytopathy
		Maas et al. (187)	1 case report after 1^st^ BNT162b2 vaccine	This case can provide support for a potential association between the BNT162b2 vaccine and onset of MCD
		Komaba et al. (188)	1 case report after 1^st^ BNT162b2 vaccine	Whether SARS-CoV-2 vaccines could trigger a relapse of MCD or other forms of nephrotic syndrome is currently unclear.
	AstraZeneca (ChAdOx1)	Leclerc et al. (194)	1 case of AKI due to MCD after 1st vaccination	This report suggests a potential relationship between MCD and the Oxford-AstraZeneca COVID-19 vaccine
		Morlidge et al. (208)	2 cases of previous MCD patients relapsing after 1st vaccination	At 2 days after vaccination, one would assume the vaccine triggered a more generalized cytokine-mediated response. Others have postulated that symptoms after 4 days represent a rapid T cell–mediated response to viral mRNA
		Anupama et al. (192)	1 case of nephrotic syndrome after 1st vaccination	The temporal profile of nephrotic syndrome after the coronavirus disease 2019 vaccination and absence of any other precipitating factors points toward the vaccine as a possible trigger
**Other autoimmune phenomena**	Pfizer (BNT162b2)	Ishay et al. (204)	8 patients presenting with *de-novo* or flares of existing autoimmune conditions	Authors observed that while immune phenomena may occur following vaccination, they usually follow a mild course and require modest therapy
		Watad et al. (203)	27 cases of immune-mediated diseases flares or new disease onset within 28-days of SARS-CoV-2 vaccination	Despite the high population exposure in the regions served by these centers, IMDs flares or onset temporally-associated with SARS-CoV-2 vaccination appear rare. Most are moderate in severity and responsive to therapy although some severe flares occurred.

## Conclusion

Immune and autoimmune AEFIs after COVID-19 vaccines are rare and mostly non-life-threatening. Their rate follows 1 out of 3 possibilities, either it is very low, or similar to the occurrence rate in the general population, or even much lower in comparison to the COVID-19 infection itself. Of AEFIs mentioned, Bell’s palsy and myocarditis seemingly have the greatest risk when it comes to the mRNA-based COVID-19 vaccines however, in addition to their rarity, the disease course is mild and full recovery is the rule. In turn, GBS and VITT/VIPITT were found to be associated mainly with the adenovirus vector based COVID-19 vaccines, still with a low rate. Addressing this sort of AEFIs, their severity, and long-term effects of the COVID-19 vaccines while fighting against vaccine hesitancy is of great importance. Doubtlessly, such efforts will decrease public concern, increase vaccination coverage rate, and guide physicians towards the rapid identification of AEFIs, reassuring patients, and applying appropriate treatment in a timely manner.

## Author Contributions

NM: Supervision, Writing- Review and Editing. NL: Writing - Original Draft, Software. AO: Writing - Original Draft. RS: Writing - Original Draft. AA: Writing - Original Draft. MA: Writing - Original Draft. MZ: Writing - Original Draft. NB: Conceptualization, Writing- Review and Editing. All authors contributed to the article and approved the subitted version.

## Conflict of Interest

The authors declare that the research was conducted in the absence of any commercial or financial relationships that could be construed as a potential conflict of interest.

## Publisher’s Note

All claims expressed in this article are solely those of the authors and do not necessarily represent those of their affiliated organizations, or those of the publisher, the editors and the reviewers. Any product that may be evaluated in this article, or claim that may be made by its manufacturer, is not guaranteed or endorsed by the publisher.
